# Piezoelectric-Assisted Removal of a Mandibular Cementoossifying Fibroma: An Innovative Technique

**DOI:** 10.1155/2020/8821090

**Published:** 2020-09-08

**Authors:** Adel Bouguezzi, Hajer Hentati, Abdellatif Chokri, Sameh Sioud, Jamil Selmi

**Affiliations:** ^1^Faculty of Dental Medicine of Monastir, Oral Health and Oro-Facial Rehabilitation Laboratory Research (LR12ES11), University of Monastir, Monastir, Tunisia; ^2^Dental Clinic of Monastir, Department of Medicine and Oral Surgery, Monastir, Tunisia

## Abstract

Diagnosis of cementoossifying fibroma is oriented by the clinical and radiological aspects of the lesion. Histology confirms the diagnosis. Treatment is surgical with enucleation-resection depending on the lesion size or wider resection with bone reconstruction in cases of large fibromas. The use of piezoelectric bone surgery is associated with low surgical trauma, exceptional precision, and fast healing response. It also allows easy performance of complex osteotomy and reduces the necessary dimensions of mucoperiosteal dissection. The purpose of the present article was to present the advantages of piezoelectric-assisted surgical removal of a cementoosseous fibroma of the mandible and to provide a precise description of the procedure using atraumatic surgery.

## 1. Introduction

Cementoossifying fibroma has been classified as ossifying fibroma or cementifying fibroma [1], but in the fourth issue of the World Health Organization (WHO) for the classification of tumors in the head and neck, published in January 2017, the term “cementoossifying fibroma (COF)” was added [2]. A successful extirpative surgery consisting in the complete removal of the infected organ or the tissue having negative margins is required. However, preservation of the major anatomical structures should always be attempted. The present report presents a technical alternative in the form of a piezoelectric surgical device for a safe and simple excision in the treatment of focal cementoossifying fibroma.

## 2. Observation

A 36-year-old female patient presented to our department for a localized right mandibular pain in the posterior area associated with swelling of the right lower jaw. This symptomatology was the main reason for her consultation. Palpation of the lymph nodes showed lymphadenopathy under the right maxilla. However, cutaneous mucosal sensitivity on the side affected by the lesion was preserved.

**Figure 1:**
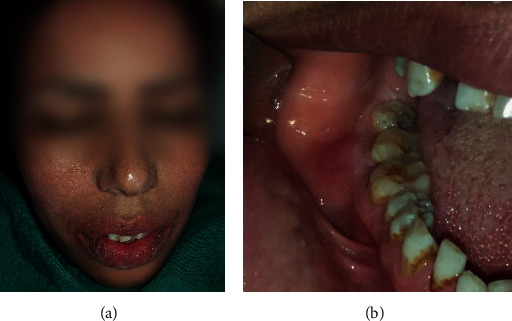
(a) Extraoral photograph. (b) Vestibular tumefaction regarding the 47; the recovering mucosa appears normal.

Intraoral examination revealed the presence of a firm vestibular swelling in the region of the 47. The fibromucosa was intact and showed a hard mass at the vestibular alveolar ridge. The surrounding mucosa was normal. The patient had defective oral hygiene. The vitality of the other teeth was retained (Figure 1).

**Figure 2:**
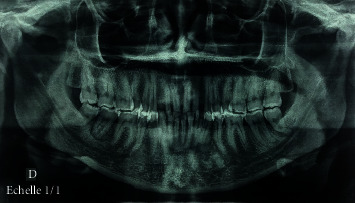
Preoperative orthopantomogram revealing a radiolucent lesion associated with radiopaque content regarding the 47.

**Figure 3:**
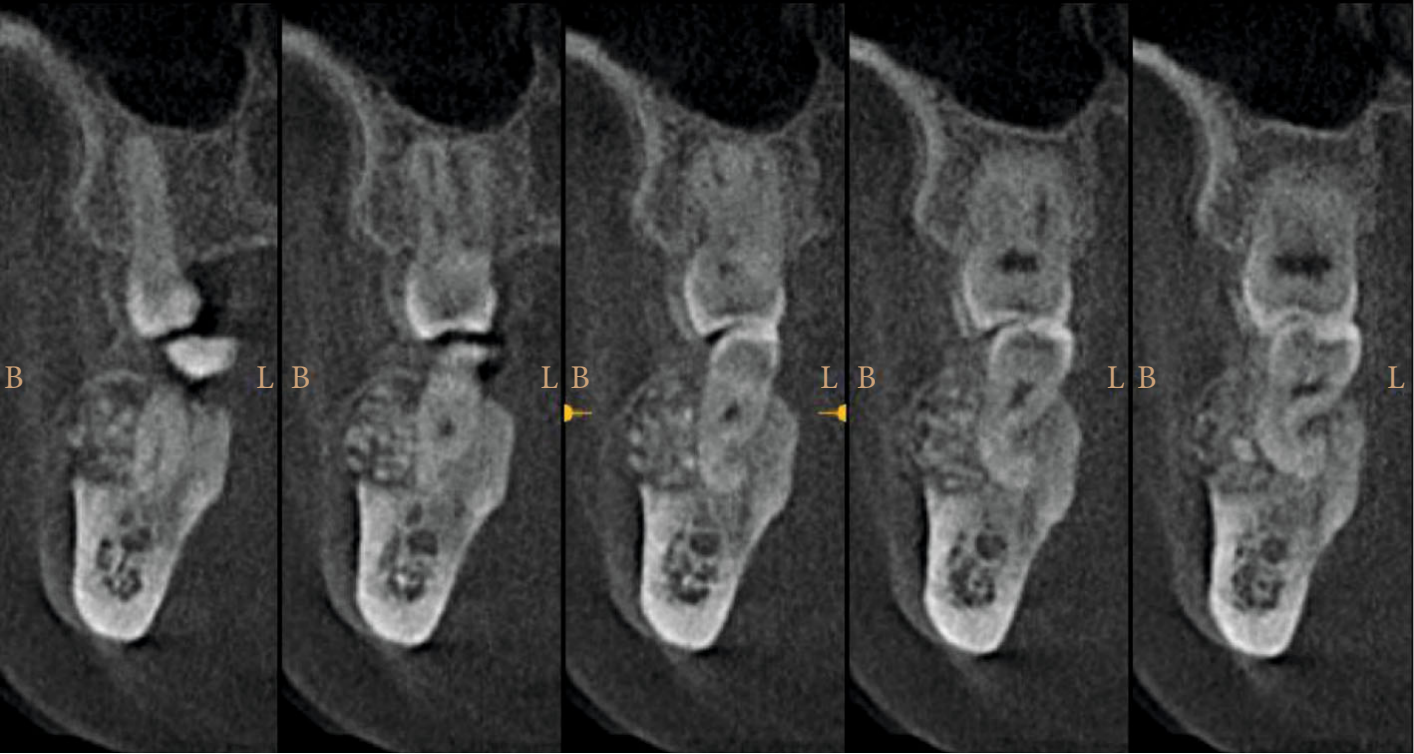
CT coronal cross slides of right posterior mandible showing expansion of the vestibular cortical.

Panoramic radiograph and CBCT revealed a well-delimited radiolucent lesion associated with radiopaque content having irregular shape, extending from the mesial root of the 48 to the distal root of the 46. The lesion was not related to the lower alveolar canal. The contour of the deformed buccal bone as well as the integrity and position of the adjacent teeth was preserved (Figures 2 and 3).

Faced with this clinical aspect, the diagnostic hypotheses were in favor of a benign odontogenic tumor of the mandible. Radiological evidence guided the diagnosis towards ossifying fibroma which was easily distinguished from diffuse sclerosing osteomyelitis, Paget's disease, or other mixed benign bone tumors. The treatment consisted in the excision of the whole tumor under local anesthesia. An intrasulcular incision at the lesion site (47), supplemented by vestibular mesial discharge, was made. The mucoperiosteal flap was elevated. Then, removal of the bone lesion was performed using the piezosurgical technique with a Piezosurgery® device (Mectron®-Germany, Cologne, Germany).

A straight insert was mounted on the piezoelectric device to axially cut the alveolar bone. Then, an angle-shape insert was mounted to make 2 vertical piezo incisions. These different inserts allowed, in a very simple and easy manner, to accede to the areas that were otherwise difficult to reach. A straight chisel was then used to complete the tumoral excision. Finally, a round-shaped cutter was utilized to smooth the remaining sharp bone edges.

**Figure 4:**
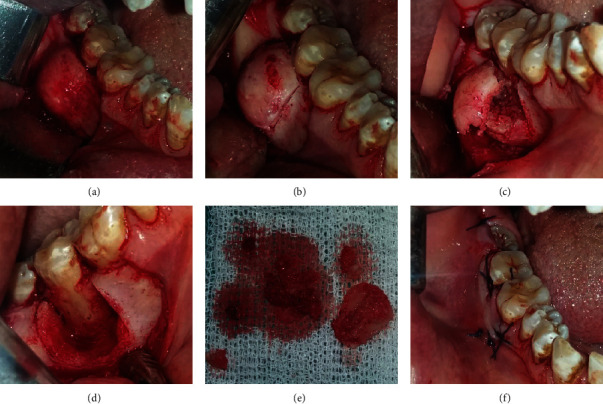
(a) Surgical access to the vestibular mandibular region showing bone deformation. (b) Minimal operative and atraumatic vertical osteotomy was performed by piezosurgery. (c) Intraoperative photograph following tumor exposure. (d) Surgical enucleation with curettage of the lesion; note the large defect and the exposition of the roots of the 47. (e) Surgical piece. (f) Flap repositioning and sutures.

The granulomatous tissues and calcifications were removed by curettage, and an abundant rinsing of the cavity was carried out with physiological serum. The excised fragments were sent to the laboratory for an anatomopathological examination which revealed the presence of irregular cementoosseous tissue. A curative antibiotic therapy, combining amoxicillin and metronidazole, was administered after repositioning the flap and sutures (Figure 4).

**Figure 5:**
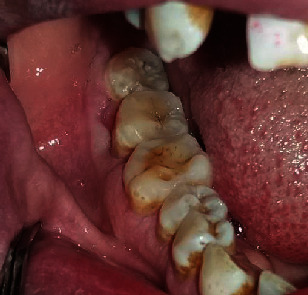
Healing 10 days postoperatively; complete healing of the soft tissue.

**Figure 6:**
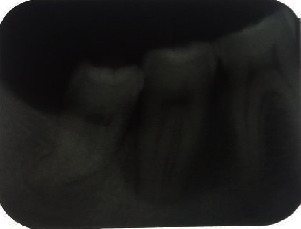
Periapical radiograph 10 days postoperatively; satisfactory radiological aspect with complete tumor removal.

Postoperative follow-up was carried out (Figures 5 and 6). At first, we tried to preserve the 47, but due to persistent mobility, it was extracted one month after the surgery. No postoperative complications were observed.

## 3. Discussion

Fibroosseous lesions include a multitude of developmental, dysplastic, and neoplastic pathologies showing diverse clinical, radiological, and histological aspects. One of their variants is cementoossifying fibroma (COF).

Ossifying fibroma (OF) is considered by some authors as the most common benign fibroosseous lesion of the maxillofacial and oral region [3].

It generally arises between the second and fourth decades of life, with a 1 : 5 male : female ratio. Its most frequent location is the mandible (3/4), involving the molar and premolar regions. The facial sinuses and nasal cavities can rarely be involved [4].

Clinically, COF usually presents as a painless spherical or ovoid expansive mass. Paresthesia or pain can be present, especially during infection. The lesion can also cause facial deformity, proptosis, sinus obstruction, and intracranial complications although it can remain asymptomatic during the first stages of development [5].

The tumor elaborates bone and spheroidal calcifications. Radiographically, it is described as a well-circumscribed, generally slow-growing, progressive, and painless bone tumefaction, with expansile, sharply defined margins, often with a radiolucent peripheral or variable radiopaque component. It is usually unilocular, but multilocular types are not uncommon. It enlarges in an expansile manner and may reach a very large size, resulting in considerable deformity [6].

Depending on its degree of mineralization, it is more or less firm in consistency. Cementoossifying fibroma is covered with normal mucosa. No general signs or associated adenopathies are present [7].

A more aggressive form of ossifying fibroma occurring in younger patients is known as juvenile osseous fibroma (JOF), being more clinically aggressive and more vascular at the pathological examination. JOF usually involves the maxilla and the paranasal sinuses, contradictory to COF which occurs in the mandible in 70–80% of cases [8, 9].

The recurrence rate of COF is reported to be 28%, and there is a remote possibility of malignant transformation. So, treatment ranges from surgical curettage or enucleation to resection and radical surgery, followed by long-term observation [4, 10, 11].

Without treatment, the lesion evolves slowly and progressively. The increasing lesion volume represents a threat to the adjacent dental roots which become mobile. Excision of the bone lesion must be complete because partial resection can maintain the infection and therefore lead to recurrence in other areas [4].

Cutting the bone without injuring the nearby soft tissues has always been a clinical challenge [12, 13]. Invasive surgeries with rudimentary instruments, such as drills, burs, and saws, have been widely used in dental clinics [14]. Yet, despite their effectiveness, they can be problematic to handle when used in proximity to soft tissues and to important anatomical structures, such as nerves and blood vessels [15, 16].

Complications related to oral surgeries of the jaws include incomplete resection, damage to nearby vital structures, severe hemorrhage, hematoma, infection, malocclusion, and iatrogenic bone fracture [17].

With the constant advancement in medicine and surgical techniques, as well as technology, all options should be considered during the operative procedure to help reduce any predictable complication risks during surgery. New surgical tools have been developed in order to minimize iatrogenic injury. The use of piezosurgery is an alternative, which uses ultrasonic vibrations to selectively cut through the bone without harming the soft tissues, thus providing a sensible use in corticotomy, especially in atypic or vulnerable anatomic regions, such as the nerve or sinus. Several studies have shown that the use of piezosurgery significantly decreases damage to the soft tissues and neurovascular bundle. It also improves bone healing when compared to bur osteotomy in simple oral surgery [18–20].

With regard to resection of odontogenic tumors, the use of piezosurgery is a contemporary approach that has been the topic of a small number of publications in the literature [21, 22]. Yaman et al. [23] revealed the advantages of piezosurgery in the protection of vital structures when the surgery is within close vicinity to those structures. Ochiai et al. [24] reported a minimal invasive procedure with endoscopy- assisted piezo surgery that drastically reduces the risk of complication and damage to the adjacent vital structures.

The use of the piezoelectric technique has also been reported to cause less postoperative pain and swelling and to reduce the recovery time [25]. It also reduces the risk of emphysema [26] and allows for increased osteotomy precision due to the predictable force and the cutting rate, contrary to conventional drills and saws that are greatly affected by the density of the mineralized tissue. In this context, the application of ultrasonic vibration and its related powerful cavitation might decrease the microbial critical mass around the involved bone and allow synergy with the medical treatment [27].

The piezosurgery technique greatly decreases the risk of damaging the soft tissues, such as sinus floor membrane, nerves, and vessels. Nevertheless, precautions must be taken as ultrasonic waves have mechanical energy, which can be converted into heat and pass into the adjacent tissues. Moreover, the main disadvantage of the procedure using the piezosurgery unit is the increased operation time required for bone preparation [28].

## 4. Conclusion

The piezoelectric device is widely applied in oral surgery. This paper presents a rare report with regard to its utility in the treatment of cementoossifying fibroma. This ultrasonic lancet is an innovation, allowing to perform a secure and precise intervention in many delicate cases. Despite its numerous potentials, it is important to define a reasoned field of application for this tool. Indeed, for an experienced practitioner, using the ultrasonic lancet may slow the surgery down in some cases because it is less invasive than the conventional techniques. Long-term, controlled randomized studies are needed to support the use of this device in oral tumor surgery.
